# Renal-clearable hyaluronic acid functionalized NaGdF_4_ nanodots with enhanced tumor accumulation†

**DOI:** 10.1039/c9ra08974h

**Published:** 2020-04-06

**Authors:** Yining Yan, Lei Ding, Lin Liu, Murad M. A. Abualrejal, Hongda Chen, Zhenxin Wang

**Affiliations:** Department of Radiology, China-Japan Union Hospital of Jilin University Xiantai Street Changchun 130033 P. R. China LiuLin99@jlu.edu.cn; State Key Laboratory of Electroanalytical Chemistry, Changchun Institute of Applied Chemistry, Chinese Academy of Sciences Changchun 130022 P. R. China chenhongda@ciac.ac.cn; School of Applied Chemical Engineering, University of Science and Technology of China Road Baohe District Hefei Anhui 230026 P. R. China

## Abstract

Integration of high tumor-targeting capacity, controlling *in vivo* transport and low normal tissue retention into one engineered nanoparticle is a critical issue for future clinically translatable anti-cancer nanomedicines. Herein, hyaluronic acid functionalized 3.8 nm NaGdF_4_ nanodots (named NaGdF_4_ ND@HAs) have been prepared through conjugation of tryptone capped NaGdF_4_ nanodots (NaGdF_4_ ND@tryptone) with hyaluronic acid (HA, a naturally occurring glycosaminoglycan), which can recognize the overexpressed CD44 on cancer cell membranes. The as-prepared NaGdF_4_ ND@HAs have good paramagnetic properties (longitudinal relaxivity (*r*_1_) = 7.57 × 10^−3^ M S^−1^) and low cytotoxicity. The *in vivo* experimental results demonstrate that the NaGdF_4_ ND@HAs can not only efficiently accumulate in mouse-bearing MDA-MB-231 tumors (*ca.* 5.3% injection dosage (ID) g^−1^ at 2 h post-injection), but also have an excellent renal clearance efficiency (*ca.* 75% injection dosage (ID) at 24 h post-injection). The as-prepared NaGdF_4_ ND@HAs have good paramagnetic properties with enhanced tumor-targeting capacity, which provides a useful strategy for the preparation of renal clearable magnetic resonance imaging (MRI) contrast agents for tumors.

## Abstract 2

Hyaluronic acid functionalized NaGdF_4_ nanodots were synthesized and evaluated as an active tumor-targeting magnetic resonance imaging (MRI) contrast agent.

## Graphical abstract

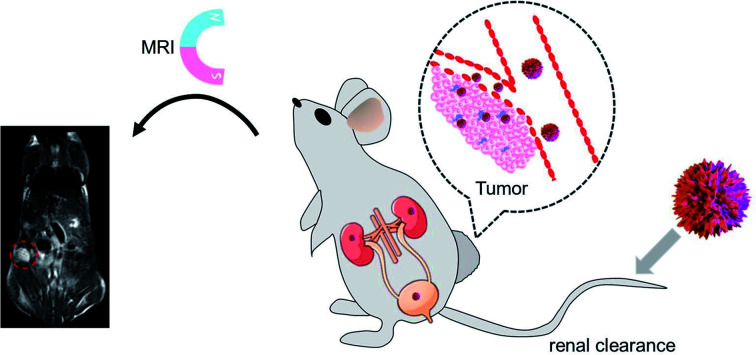

## Introduction

As one of the prospective directions of contemporary medical sciences, nanomaterial-based theranostics (known as nanomedicines) have been intensively studied because of their potential applications in various domains including diagnosis of and therapies for diseases (*e.g.*, tumor), tracking the location of biomarkers/signal molecules *in vivo*, and evaluating their therapeutic effect.^1–10^ To date, more than one hundred nanomedicines have been approved by the Food and Drug Administration (FDA) in the USA, or are in the FDA clinical trial stage.^4^ However, it is a great challenge to translate nanomedicines from pre-clinical proof-of-concept to clinical applications because there is an increasing consideration of the biosafety of nanomedicines.^11–18^ Currently, many promising nanomedicines normally eliminated through the hepatobiliary route are constructed from relatively large sized (>10 nm in diameter) and nonbiodegradable inorganic nanoparticles.^16–19^ However, the interactions of nanomedicines with mononuclear phagocyte system (MPS) cells (*e.g.*, Kupffer cells) in spleen and liver significantly increase their retention time *in vivo* (even more than months), which causes potential toxicity.^11–19^ The biosafety concern can be addressed by the development of nanomedicines that undergo renal clearance.^20–22^ During the last few years, several ultra-small sized inorganic nanoparticles (<5.5 nm) have been employed to generate renal clearable nanomedicines for the diagnoses and therapies of various diseases including cancers.^23–33^ For instance, Zheng's group has been developed a series of gold nanoparticles/nanoclusters for delivering anticancer drugs,^30^ evaluating kidney function,^31^ and imaging tumors.^32,33^

As a powerful tool, magnetic resonance imaging (MRI) has been extensively applied for non-invasive diagnoses of various diseases through producing excellent soft-tissue contrast. Compared with other modalities of medical imaging, the sensitivity of MRI is poor. Because trivalent gadolinium ion (Gd^3+^) has seven unpaired electrons with a large magnetic moment, Gd-chelates and Gd nanoparticles have been employed as contrast agents for enhancing the sensitivity of *T*_1_-weighted MRI.^34–38^ Among Gd-based *T*_1_-weighted MRI contrast agents, ultrasmall Gd nanoparticles (also known as Gd nanodots (Gd NDs)) not only have high contrast enhancement capability, but also can be eliminated from body through renal clearance.^39–47^ The Gd NDs are normally coated hydrophilic or amphiphilic ligands for improving their colloidal stability and biocompatibility, then further modified with specific biomolecules for generating high tumor-targeting ability. As a major component of extracellular matrices, hyaluronic acid (hyaluronan, HA) has high binding affinity with cell surface receptor, CD44.^48–50^ Therefore, HA can be used as an active tumor-targeting ligand for constructing drug delivery systems since tumor cells normally express high level of CD44.^50–54^ For example, Parayath and coauthors have fabricated hyaluronic acid–poly(ethylenimine) (HA–PEI)-based nanoparticles encapsulating miR-125b for anticancer immunotherapy through the interactions of HA with CD44.^53^ In addition, Guo *et al.* have found that Gd^3+^-labeled peptide dendron-HA conjugate-based hybrid (dendronized-HA-DOTA-Gd) has better biocompatibility and higher accumulation in tumors than those of Gd-DTPA.^51^ The results suggest that HA might be employed as an active tumor-targeting ligand for generating renal clearable Gd NDs.

In this study, a highly renal-clearable HA functionalized NaGdF_4_ nanodots (NaGdF_4_ ND@HAs) were synthesized by a two-step reaction, and evaluated as an active tumor-targeting MRI contrast agent. The tryptone was employed as phase transfer agent for transferring hydrophobic oleic acid coated NaGdF_4_ nanodots (NaGdF_4_ ND@OAs, 3.8 nm in diameter) through the Gd–phosphate coordination reaction. HA was then conjugated with the tryptone coated NaGdF_4_ NDs (NaGdF_4_ ND@tryptone) by the use of 1-ethyl-3-(3-dimethylaminopropyl)carbodiimide (EDC) as coupling agent. Both *in vitro* cellular studies and *in vivo* small animal experiments of mouse-bearing MDA-MB-231 breast cancer model demonstrated that NaGdF_4_ ND@HAs exhibit low toxicity and can be used for specific detection of tumors with abundant HA receptor.

## Experimental section

### Materials

Tryptone was purchased from Oxoid Ltd (Hampshire, England). Oleic acid (OA, 90%), 1-octadecene (ODE, 90%) were obtained from Sigma-Aldrich Co. (St. Louis, USA). The GdCl_3_·6H_2_O was purchased from Beijing HWRK Chem Co. Ltd (Beijing, China). The culture medium Leibovitz L-15 and DMEM were purchased from Jiangsu KeyGen Biotech Co. Ltd (Jiangsu, China). The fetal bovine serum (FBS) was obtained from Gibco Co. (New York, USA). Trypsin–EDTA digest and 3-(4,5-dimethyl-2-thiazolyl)-2,5-diphenyl-2-*H*-tetrazolium bromide (MTT) were purchased from Beijing Dingguo Biotechnology Ltd (Beijing, China). Hyaluronic acid (HA, 4.2 kDa) was purchased from Bloomage Freda Biopharma Co., Ltd (Jinan, China). Other reagents (analytical grade) were purchased from Beijing Chemical Reagent Co. (Beijing, China), which were used without any purification. Deionized H_2_O (18.2 MΩ cm) were used in all experiments. All animal procedures were carried out with the procedures approved by the local Animal Research Committee at Jilin University (ethical approve document number: 2019515). Female Balb/c nude mice and Balb/c mice (*ca.*, average body weight of 20 g) were purchased from Liaoning Changsheng Biotechnology Ltd (Liaoning, China). The mice having free access to food and water were kept for 12 h in light and 12 h in dawn daily at 20 °C.

### Characterizations

TEM micrographs were recorded by TECNAI G2 high-resolution TEM (FEI Co., USA). Dynamic light scattering and zeta potential of the as-prepared samples were carried out on a Zetasizer Nano ZS (Malvern Instruments Ltd, UK). The analysis of elements was conducted with an ELAN 9000/DRC ICP-MS system (PerkinElmer, USA). The relaxation times of the samples were carried out on a Siemens Prisma 3.0 T MR scanner (Erlangen, Germany). Energy-dispersive X-ray spectra (EDS) were inspected on an energy dispersive spectroscopy (FEI Co., USA). X-ray diffraction analysis were carried out on a D8 ADVANCE diffractometer (Bruker Co., Germany) using Cu Kα (0.15406 nm) radiation. The infrared spectra were conducted with a Vertex 70 Fourier transform infrared (FTIR) spectrometer (Bruker, Germany). XPS measurements were conducted with a VG ESCALAB MKII X-ray photoelectron spectrometer (VG Scientific Ltd, UK) spectroscopy (XPS). Siemens Prisma 3.0 T MR scanner (Erlangen, Germany) was employed to acquire *T*_1_-weighted MR images.

### Synthesis of NaGdF_4_ ND@HAs

The NaGdF_4_ ND@OAs were synthesized by previously reported methods (see ESI† for details).^40,43,47^ Subsequently, 10 mL NaGdF_4_ ND@OA cyclohexane solution (1 mg mL^−1^) were mixed with 10 mL tryptone aqueous solution (6.4 mg mL^−1^) under vigorously stirred, and incubated at room temperature for 12 h. Then, the as-prepared tryptone capped NaGdF_4_ nanodots (NaGdF_4_ ND@tryptone) were purified by centrifugation (13 000 rpm, 3 times) and redispersed in H_2_O. 0.5 mL 1-ethyl-3-(3-(dimethylaminopropyl)carbodiimide solution (EDC, 4 mg mL^−1^ in MES (2-(*N*-morpholino)ethanesulfonic acid) buffer) and 0.5 mL *N*-hydroxysulfo-succinimide solution (sulfo-NHS, 6 mg mL^−1^ in MES buffer) were mixed with 2 mL HA solution (2.5 mg mL^−1^ in MES buffer). After incubation at room temperature for 1 h, 2 mL NaGdF_4_ ND@tryptone (2.5 mg mL^−1^) in PBS (phosphate buffer saline, pH 7.4)) were added into the mixture and continuously incubated at 37 °C for 3 h. Finally, the as-prepared NaGdF_4_ ND@HAs were purified by centrifugation (10 000 rpm, 3 times) and redispersed in PBS (pH 7.4).

### Cytotoxicity study

The MDA-MB-231 breast cancer cells, MCF-7 breast cancer cells and normal kidney tissue cells (293 cells) were obtained from Shanghai Cell Bank, CAS (Shanghai, China). MDA-MB-231 breast cancer cells were cultured with fresh L-15 culture medium supplemented with 10% FBS in a 96 well plate (1 × 10^4^ cells in 100 μL per well) under full air conditions at 37 °C for 24 h, MCF-7 breast cancer cells and normal kidney tissue cells (293 cells) were cultured with fresh DMEM culture medium supplemented with 10% FBS and 100 U mL^−1^ penicillium–streptomycin in a 96 well plate (1 × 10^4^ cells in 100 μL per well) under 5% CO_2_ air conditions at 37 °C for 24 h. Subsequently, those cells were washed by PBS. Next, 100 μL fresh culture medium containing various concentrations (6.25, 12.5, 25, 50, 100 and 200 μg mL^−1^) of NaGdF_4_ ND@tryptone or NaGdF_4_ ND@HAs were introduced into wells, and cultured for another 24 h. After fully washed by PBS (100 μL, 3 times), the cell viabilities were detected by traditional MTT (3-(4,5-dimethyl-2-thiazolyl)-2,5-diphenyl-2-*H*-tetrazolium bromide) assay.

### *In vitro* MRI study

The MDA-MB-231 and MCF-7 cells were cultured at 37 °C for 24 h and divided into 4 groups ((1) MCF-7 + NaGdF_4_ ND@HAs; (2) free HAs (2 mg mL^−1^) + MDA-MB-231 incubated for 4 h, and then incubated with NaGdF_4_ ND@HAs; (3) MDA-MB-231 + NaGdF_4_ ND@tryptone; (4) MDA-MB-231 + NaGdF_4_ ND@HAs), which were co-cultured with different concentrations of NaGdF_4_ ND@tryptone or NaGdF_4_ ND@HAs in 2 mL fresh culture medium containing different concentrations of NaGdF_4_ NDs at 37 °C for 24 h, respectively. After washed with fresh culture medium (1 mL, 3 times) and PBS (1 mL, 3 times), the NaGdF_4_ ND stained cells were digested by 1 mL trypsin and centrifuged at 2000 rpm for 5 min. Subsequently, the as-obtained cell pellets were fixed by 1% agarose gel, and imaged by a Siemens 1.5 T MRI scanner with imaging parameters: 1.2 mm slice thickness, 15 ms echo time (TE), 358 ms repetition time (TR) and 50 mm × 50 mm field of view. In addition, 3 × 10^3^ NaGdF_4_ ND stained cells were treated by 2 mL aqua regia. The amounts of Gd element in the NaGdF_4_ ND stained cells were measured by inductively coupled plasma mass spectrometry (ICP-MS, ELAN 9000/DRC, PerkinElmer Co., USA), respectively.

### *In vivo* MRI study

Female Balb/c nude mice and Balb/c mice with average body weight of 20 g were purchased from Liaoning Changsheng Biotechnology Ltd (Liaoning, China). The mice having free access to food and water were kept for 12 h in light/12 h in dawn daily at 20 °C. All animal procedures were approved by the Local Ethics Committee for Institutional Animal Care and Use of Jilin University. MDA-MB-231 cells (1 × 10^6^ cells in 100 μL PBS) were subcutaneously injected into the hind flank of female Balb/c nude mouse. The volume of tumor (*V*) was evaluated by the following formula: *V* = length × (width)^2^/2. When *V* reached about 60 mm^3^, the MDA-MB-231 tumor-bearing nude mice were injected intravenously with 100 μL 0.9 wt% NaCl solution containing 2 mg mL^−1^ (Gd^3+^ content) NaGdF_4_ ND@tryptone or NaGdF_4_ ND@HAs through tail veins. The *in vivo* MR images of tumors were taken at 0, 0.5, 2, 4, 8, 12, and 24 h post-injection by Siemens 3.0 T MRI scanner with the following scanning parameters: 1.2 mm slice thickness, 3000 ms TR, 9.1 ms TE and 120 mm × 72 mm field of view. In addition, the mice were sacrificed at 2 h post-injection, and the main organs as well as tumors were collected for ICP-MS measurement.

### Biocompatibility analysis

The healthy female Balb/c mice were randomly divided into 2 groups: control group and NaGdF_4_ ND@HAs treated group. The mice in treated group were injected intravenously with 100 μL 0.9 wt% NaCl solution containing 10 mg kg^−1^ (Gd^3+^ content) NaGdF_4_ ND@HAs, respectively. The mice in control group were only injected intravenously with 100 μL 0.9 wt% NaCl solution respectively. The body weights of mice were measured every 2 days until 30 days after injection. The mice were sacrificed at the 1^st^ and the 30^th^ day post-injection, and main organs including heart, liver, spleen, lung and kidneys were fixed in 4% (w/v) paraformaldehyde solution, embedded in paraffin, sectioned, and finally stained with hematoxylin–eosin (H&E). Meanwhile, the tumors were fixed in 4% (w/v) paraformaldehyde solution, embedded in paraffin, sectioned, and finally stained by hematoxylin–eosin (H&E) and anti-CD44v6 immunohistochemistry. The blood samples of mice were collected at the 1^st^ and the 30^th^ day post-injection, also analysed by the blood routine assay.

## Results and discussion

### Synthesis and characterization of NaGdF_4_ ND@HAs

**Scheme 1:**
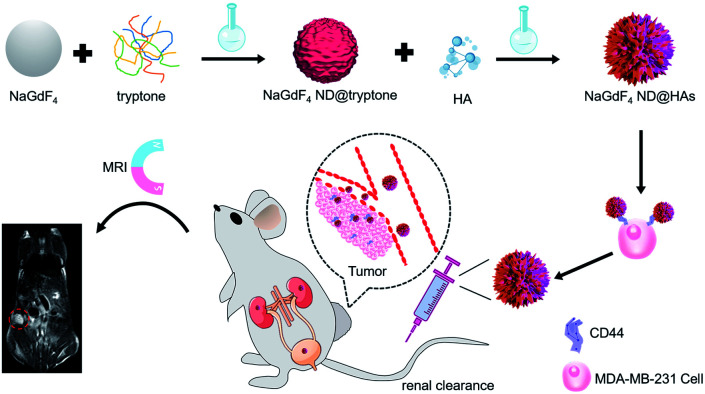
Schematic diagram of NaGdF4 ND@HAs synthesis, and the application in MRI of tumor through recognizing the overexpressed CD44 on cancer cell membrane.

**Fig. 1:**
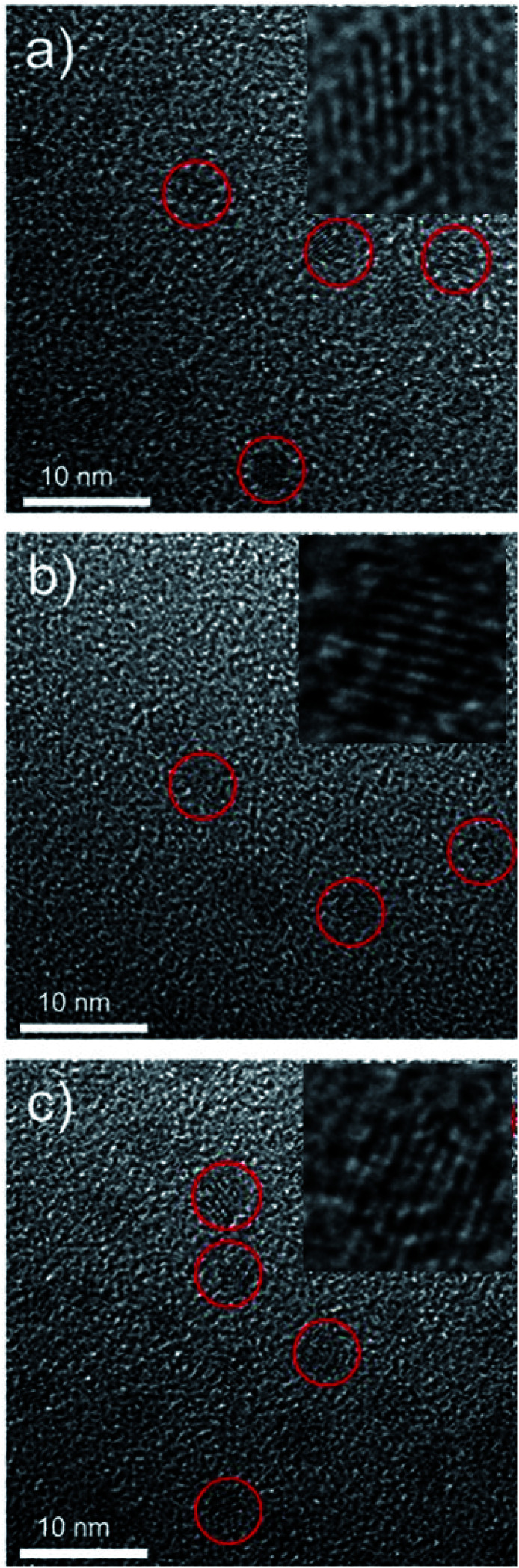
Transmission electron microscope micrographs (TEM) of (a) NaGdF_4_ ND@OAs, (b) NaGdF_4_ ND@tryptone and (c) NaGdF_4_ ND@HAs, respectively. Insets are corresponding HRTEM micrographs of NaGdF_4_.

**Fig. 2:**
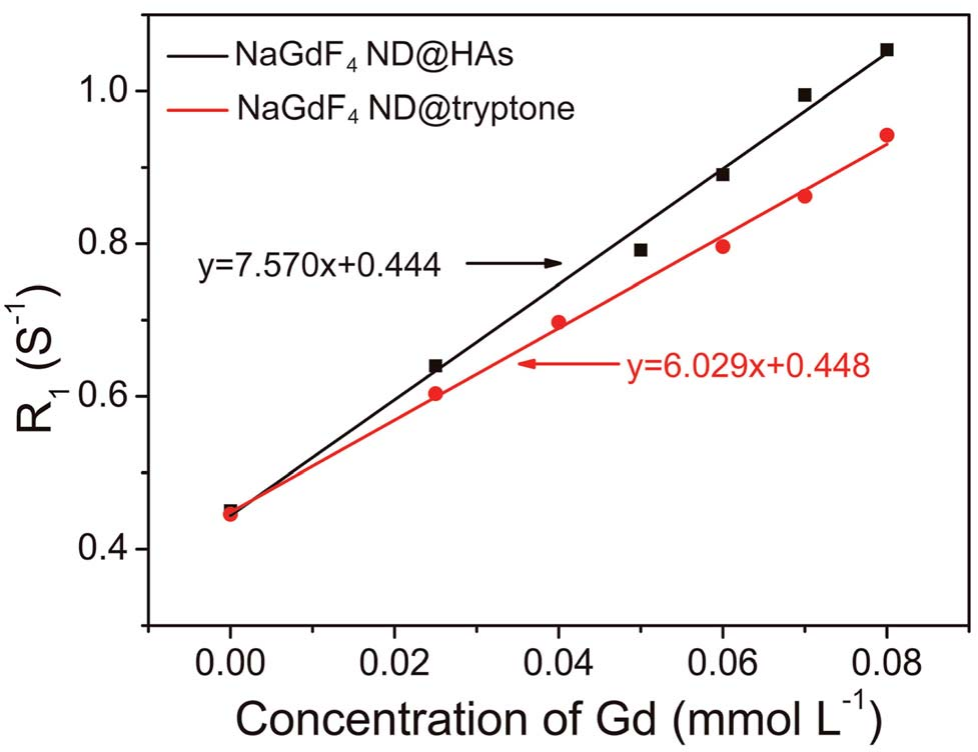
*R*_1_ relaxivities of NaGdF_4_ ND@tryptone and NaGdF_4_ ND@HAs as a function of the molar concentration of Gd^3+^ in solution, respectively. The slope of line represents the longitudinal relaxivity (*r*_1_) value.

The synthetic route and application of NaGdF_4_ ND@HAs is shown in Scheme 1. The hydrophobic NaGdF_4_ ND@OAs (3.8 ± 0.4 nm in diameter) were prepared by previously reported procedure with a slight modification (as shown in Fig. 1a).^40,43,47^ As the digestion product of casein, tryptone contains *ca.* 10–20% casein phosphopeptide (CPP) with the sequence –Ser(P)–Ser(P)–Ser(P)–Glu–Glu–, which can form robust Gd^3+^–phosphate coordination bonds under mild conditions through the reaction of phosphoseryl serine residue (Ser(P)) and trivalent Gd ions.^43,47^ After mixing NaGdF_4_ ND@OAs with tryptone, hydrophilic NaGdF_4_ ND@tryptone were generated through replacing the original OA ligand of NaGdF_4_ ND@OAs by tryptone. Subsequently, HA was activated by EDC and sulfo-NHS, also conjugated on NaGdF_4_ ND@tryptone surface through the amidation reaction between carboxy group of HA and amine group of tryptone. HA and tryptone, which have been extensively used for producing drugs and health care products, are raw materials approved by the US Food and Drug Administration (FDA). Therefore, the toxicity of NaGdF_4_ ND can be reduced by HA and tryptone coating. After ligand exchange and HA functionalization, the morphology, size and crystalline nature of NaGdF_4_ NDs exhibit negligible changes (as shown in Fig. 1b and c). The hydrodynamic diameter and zeta potential of NaGdF_4_ ND@tryptone were 11.99 nm and −5.86 mV, respectively. The result was consistent with the structure of NaGdF_4_ ND@tryptone which contains individual solid NaGdF_4_ ND core and a flexible phosphopeptide outlayer. The phenomenon suggested that NaGdF_4_ ND@tryptone exhibits good monodispersity and negative surface charge. Because HA is negatively charged biomacromolecule, the hydrodynamic diameter of NaGdF_4_ ND@HAs was increased to 29.05 nm, while the zeta potential of NaGdF_4_ ND@HAs was deceased to −12.16 mV. The negative surface charge helps to reduce the nonspecific interactions of NaGdF_4_ NDs with cells. The successful preparation of NaGdF_4_ ND@tryptone and NaGdF_4_ ND@HAs were also investigated by XPS, EDS and FTIR. After incubation with tryptone, the phosphorus and nitrogen peaks were clearly observed in the XPS (P 2p (133 eV) and N 1s (400 eV)) and EDS (P (2.01 keV) and N (0.39 keV)) spectra of NaGdF_4_ NDs (as shown in Fig. S1 and S2†).^43,47,55^ In addition, the XPS spectrum of NaGdF_4_ ND@HAs exhibited relatively high intensity of C 1s (284 eV). Compared to NaGdF_4_ ND@OAs, two additional IR bands at 683 cm^−1^ and 1080 cm^−1^ are observed in FTIR spectrum of NaGdF_4_ ND@tryptone (as shown in Fig. S3†), which are corresponded to the out-of-plane bending vibration of C–H bond on benzene ring and antisymmetric bending mode of PO_4_^3−^, respectively.^56^ A new IR band at 1010 cm^−1^ is observed in FTIR spectrum of NaGdF_4_ ND@HAs (as shown in Fig. S3†), which is corresponded to stretching vibration of C–O band of primary alcohol of HA.^57^ As shown in Fig. 2, the longitudinal relaxivity (*r*_1_) value (7.57 mM^−1^ S^−1^) of NaGdF_4_ ND@HAs is higher than those of NaGdF_4_ ND@tryptone (6.03 mM^−1^ S^−1^) as well as commercial Gd^3+^ chelates (*e.g.*, (4.3 mM^−1^ S^−1^)). The high *r*_1_ value of NaGdF_4_ ND@HAs may stem from the strong polarity of HA molecule which may cause a spatial agglomeration of water around the Gd^3+^, leading to boost the relaxivity to high value. The result indicates that NaGdF_4_ ND@HAs can be used as efficient *T*_1_-weighted MRI contrast agent.

### The interactions of NaGdF_4_ ND with living cells

**Fig. 3:**
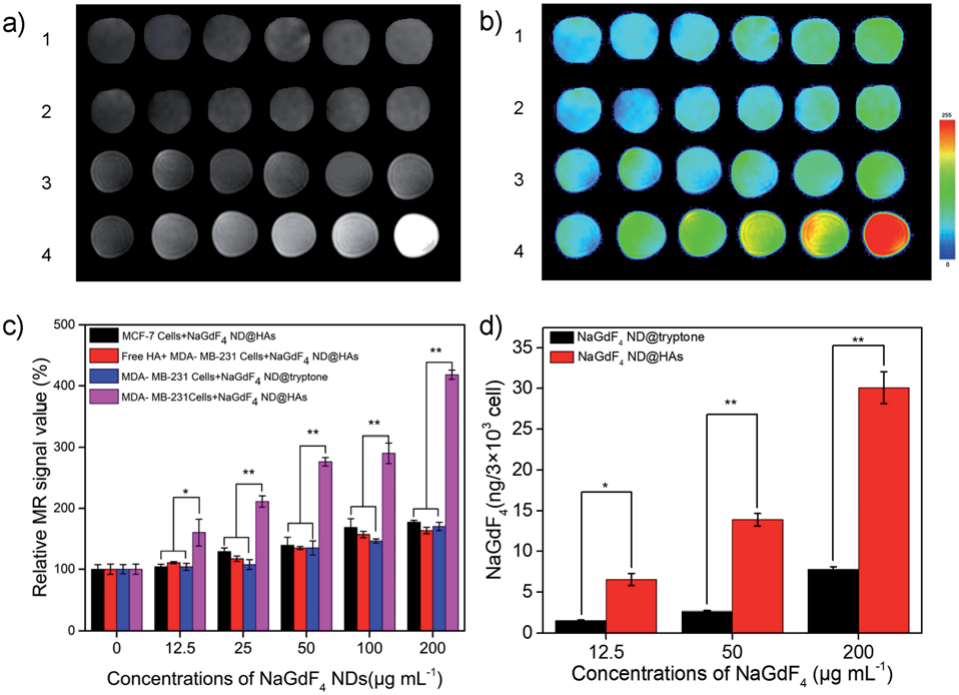
(a) MR images of NaGdF_4_ ND stained cells. ((1) MCF-7 cells + NaGdF_4_ ND@HAs; (2) free HA + MDA-MB-231 cells + NaGdF_4_ ND@HAs; (3) MDA-MB-231 cells + NaGdF_4_ ND@tryptone; (4) MDA-MB-231 cells + NaGdF_4_ ND@HA), (b) corresponding pseudo-color images and (c) corresponding data analysis of NaGdF_4_ ND stained cells. The cells were incubated with 0, 12.5, 25, 50, 100, 200 μg mL^−1^ NaGdF_4_ ND@tryptone or NaGdF_4_ ND@HAs from left to right, respectively. (d) The amounts of Gd element in the NaGdF_4_ ND stained cells. Error bars mean standard deviations (*n* = 5, *P < 0.05 or **P < 0.01 from an analysis of variance with Tukey's post-test).

Human triple-negative breast carcinoma MDA-MB-231 cell line was selected as a typical model because it expresses high level of CD44.^58^ As shown in Fig. S4,† the MDA-MB-231, MCF-7 and 293 cells exhibit higher than 90% viability after incubated with up to 200 μg mL^−1^ NaGdF_4_ ND@HAs or NaGdF_4_ ND@tryptone for 24 h. The result indicates that both of NaGdF_4_ ND@HAs and NaGdF_4_ ND@tryptone have low cytotoxicity. The *T*_1_-weighted MR signal intensity of NaGdF_4_ ND@HAs stained MDA-MB-231 cells was stronger than which of NaGdF_4_ ND@tryptone stained MDA-MB-231 cells or NaGdF_4_ ND@HAs stained MCF-7 (as shown in Fig. 3a–c). The *T*_1_-weighted MR signal intensity (Fig. 3, group 2) of NaGdF4 ND@HAs stained HA treated MDA-MB-231 cells is much lower than which of NaGdF4 ND@HAs stained untreated MDA-MB-231 cells (Fig. 3, group 4). In addition, the cellular internalization amount of NaGdF_4_ ND@HAs is much higher than which of NaGdF_4_ ND@tryptone (as shown in Fig. 3d). The results demonstrate that the NaGdF_4_ ND@HAs have high affinity with MDA-MB-231 cells, and can be used to recognize CD44-overexpression cells with high specificity.

### *In vivo* MRI of MDA-MB-231 tumor

**Fig. 4:**
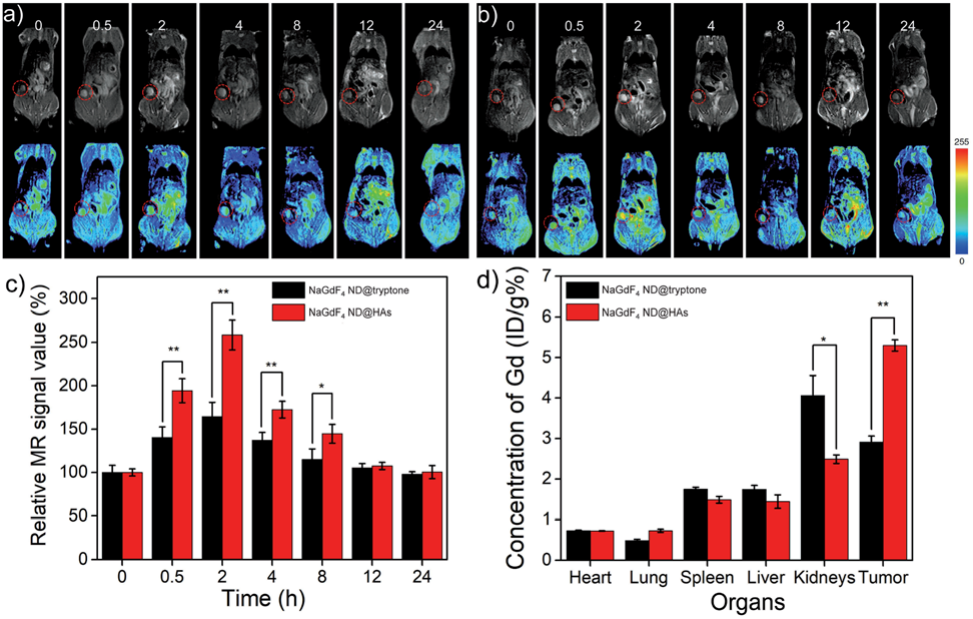
*In vivo* MR images and corresponding pseudo color images of Balb/c mouse bearing MDA-MB-231 tumor after intravenous injection of (a) NaGdF_4_ ND@tryptone or (b) NaGdF_4_ ND@HAs (10 mg Gd kg^−1^ body) at different timed intervals (0 (pre-injection), 0.5, 2, 4, 8, 12 and 24 h post-injection), respectively. (c) Corresponding data analysis of MR measurements. The tumor site was marked by circle. (d) The amounts of NaGdF_4_ NDs in main organs at 2 h post-injection. Error bars mean standard deviations (*n* = 5, *P < 0.05 or **P < 0.01 from an analysis of variance with Tukey's post-test).

A Balb/c nude mouse bearing MDA-MB-231 tumor model was established for evaluating the active tumor-targeting capacity of NaGdF_4_ ND@HAs. Both of NaGdF_4_ ND@HAs and NaGdF_4_ ND@tryptone (10 mg Gd kg^−1^ body weight in 0.9 wt% NaCl) were intravenously injected into the mouse bearing MDA-MB-231 tumor through tail vein and the MR signals of tumor sites were recorded at desired timed intervals within 24 h post-injection. The NaGdF_4_ ND@tryptone can be accumulated in tumor site through enhanced permeability and retention (EPR) effect (*i.e.*, passive tumor-targeting), while NaGdF_4_ ND@HAs can be accumulated in tumor site by EPR effect and binding of HA with CD44 (*i.e.*, active tumor-targeting). As expected, the MR signals of the tumor sites gradually increased by numbers in between 0 and 2 h post-injection (as shown in Fig. 4). Maximum MR contrast enhancement is obtained at 2 h post-injection of NaGdF_4_ NDs. In particular, the MR signal intensity in tumor site of NaGdF_4_ ND@HAs treated mouse is higher than which of NaGdF_4_ ND@tryptone treated mouse at the same post-injection time point. The maximum MR contrast enhancement in tumor site of NaGdF_4_ ND@HAs treated mouse is 1.6 times higher than which of the NaGdF_4_ ND@tryptone treated mouse. The phenomenon may due to high binding affinity of HA with over expressed CD44 on MDA-MB-231 cells. The result demonstrated that the as-prepared NaGdF_4_ ND@HAs can be severed as an excellent *T*_1_-weighted MRI contrast agent for detection of CD44-overexpression tumors (*e.g.*, triple-negative breast cancer). The active tumor-targeting capacity of NaGdF_4_ ND@HAs was also confirmed by ICP-MS measurement. As shown in Fig. 4d, the amounts of Gd in kidneys and tumors were relatively higher than in other organs, indicating that both NaGdF_4_ ND@tryptone and NaGdF_4_ ND@HAs can be efficiently accumulated in tumor sites and excreted by renal clearance. In particular, the Gd content in tumor of NaGdF_4_ ND@HAs treated mouse treated mouse was 1.87 times higher than which of NaGdF_4_ ND@tryptone treated mouse. The result indicated that the accumulation amount of NaGdF_4_ ND@HAs in tumor site is clearly improved through EPR effect as well as recognition of HA receptors of tumor cells.

### *In vivo* biodistribution and toxicology of NaGdF_4_ ND@HAs

**Fig. 5:**
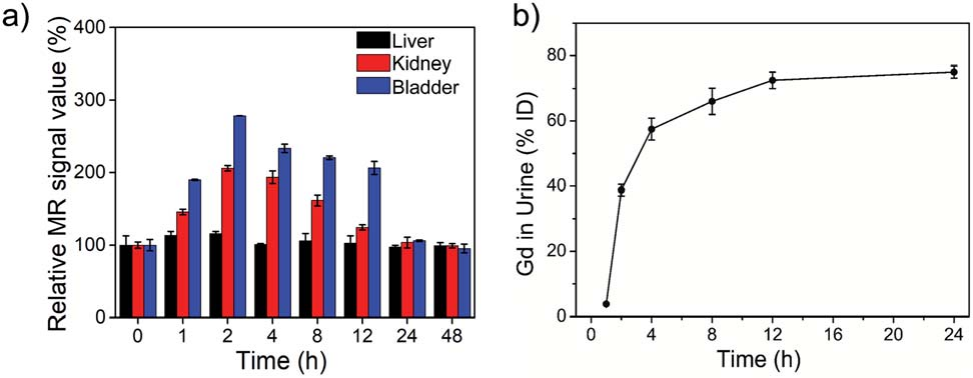
(a) The MR signal intensities of liver, kidney and bladder of healthy Balb/c mice after intravenous injection of NaGdF_4_ ND@HAs at different time intervals (0 (pre-injection), 1, 2, 4, 8, 12, 24 and 48 h) of post-injection, respectively. (b) The total amounts of NaGdF_4_ ND@HAs in mouse urine as a function of post-injection times. Error bars mean standard deviations (*n* = 5).

For evaluating its biodistribution and clearance pathway, NaGdF_4_ ND@HAs (10 mg Gd kg^−1^ body weight in 0.9 wt% NaCl) were injected into healthy Balb/c mice through tail vein. The MR signals in the liver, kidneys and bladder were recorded at different timed intervals of post-injection (as shown in Fig. 5a and S5†). The MR signal of liver exhibited little change during the whole period, indicating low accumulation of NaGdF_4_ ND@HAs in liver. MR signals in the kidneys and bladder were clearly enhanced within 24 h post-injection, and recovered to pre-injection levels after 24 h post-injection. The result demonstrated that NaGdF_4_ ND@HAs are excreted from the body by renal clearance. After intravenous injection of NaGdF_4_ ND@HAs, the Gd content in urine of mouse was measured for addressing the pharmacokinetics behavior of NaGdF_4_ ND@HAs (as shown in Fig. 5b). The total of Gd element in urine of NaGdF_4_ ND@HAs treated mouse increased significantly from 0 to 12 h post-injection. About 75% Gd was found in urine after 24 h administration, confirming that NaGdF_4_ ND@HAs were efficiently excreted with the urine. In addition, the NaGdF_4_ ND@HAs showed a negligible morphology change after *in vivo* circulation, which indicated that the NaGdF_4_ ND@HAs have good colloidal stability *in vivo* (as shown in Fig. S6†). The efficient renal clearance of NaGdF_4_ ND@HAs helped to eliminate potential hazards of long-term *in vivo* toxicity.

**Fig. 6:**
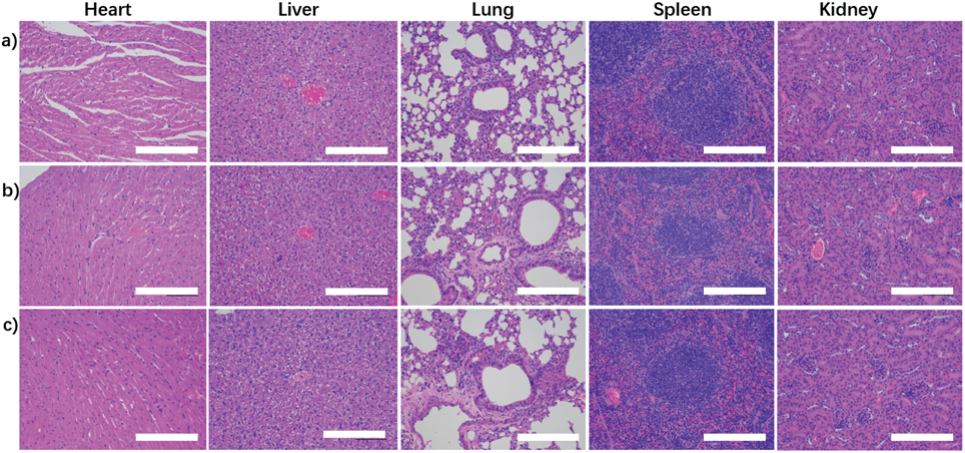
H&E staining revealed tissue sections from the mice with (a) 0.9% NaCl solution (control) and NaGdF_4_ ND@HAs (10 mg Gd kg^−1^ body) at (b) 1 day and (c) 30 days post-injection respectively. The scale bars are 200 μm.

For further evaluating the biocompatibility, the healthy Balb/c mice were intravenously injected at a single dose of NaGdF_4_ ND@HAs (10 mg Gd kg^−1^ body weight in 0.9 wt% NaCl). The bodyweights of NaGdF_4_ ND@HAs treated mice increased steadily as the time prolonged, which was consistent with those of control group (as shown in Fig. S7†). The result suggested that NaGdF_4_ ND@HAs have little effect on the growth and development of mice. The main organs (heart, liver, spleen, lung and kidneys) were collected for histology analysis at the 1^st^ day and the 30^th^ day post-injection. Comparing with the control group, the main organs of NaGdF_4_ ND@HAs treated mice showed negligible lesions or abnormalities (as shown in Fig. 6). Tumor tissue from the mice were collected for H&E staining and anti-CD44v6 staining, respectively. The experimental result indicated that cell membrane surface receptor CD44 was overexpressed on the tumor tissue (as shown in Fig. S8†). Hematology analysis was carried out for monitoring acute and long-term toxicity of NaGdF_4_ ND@HAs at the 1^st^ day and the 30^th^ day post-injection, respectively. There was little difference between NaGdF_4_ ND@HAs treated group and control group (as shown in Table S1†). These results demonstrated that NaGdF_4_ ND@HAs have good biocompatibilities.

## Conclusions

In summary, the renal clearable NaGdF_4_ ND@HAs have been prepared by a two-step reaction through strong interaction of Gd^3+^ with phosphonate groups in tryptone and amidation reaction between carboxy group of HA and amine group of tryptone. *In vitro* and *in vivo* experimental results demonstrate that the as-prepared NaGdF_4_ ND@HAs display high MDA-MB-231 tumor-targeting capacity. The NaGdF_4_ ND@HAs have held great potential as an excellent MR contrast agent for detection CD44-overexpression tumor since advantages of NaGdF_4_ ND@HAs including high tumor targeting ability, efficient renal clearance capacity and excellent biocompatibility satisfied the basic standards of clinical applications.

## Conflicts of interest

There are no conflicts to declare.

## Supplementary Material

RA-010-C9RA08974H-s001
