# Supplementary material for: Renal-clearable hyaluronic acid functionalized NaGdF4 nanodots with enhanced tumor accumulation
Source: RSC Adv. 2020 Apr 6;10(23):13872–8. doi: 10.1039/c9ra08974h (PMC9051644; doi:10.1039/c9ra08974h)
Supplement: RA-010-C9RA08974H-s001 [file RA-010-C9RA08974H-s001.pdf]

Electronic Supplementary Material (ESI) for RSC Advances.
This journal is © The Royal Society of Chemistry 2020

Electronic supplementary information for

Renal-Clearable Hyaluronic Acid Functionalized $NaGdF_4$ Nanodots with Enhanced Tumor Accumulation

Yining Yan[a,b], Lei Ding[a], Lin Liu*[a], Murad M.A. Abualrejal[b,c], Hongda Chen*[b,c] and Zhenxin Wang[b].

[a]Department of Radiology China-Japan Union Hospital of Jilin University, Xiantai Street, Changchun, 130033, P. R. China

[b]State Key Laboratory of Electroanalytical Chemistry, Changchun Institute of Applied Chemistry, Chinese Academy of Sciences, Changchun, 130022, P. R. China.

[c]School of Applied Chemical Engineering, University of Science and Technology of China, Road Baohe District, Hefei, Anhui, 230026, P. R. China.

*Corresponding Authors, *E-mails: LiuLin99@jlu.edu.cn(LL), chenhongda@ciac.ac.cn (HC).

1 Additional Experimental Section

2 Additional Figures

3 Additional Tables

4 Additional References

1 Additional Experimental Section

Synthesis of $NaGdF_4$ ND@OAs

The $NaGdF_4$ ND@OAs were synthesized by previously reported methods.[S1-S3] In brief, 1.5 mmol $GdCl_3$ aqueous solution was added into a three-neck 100 mL round-bottom flask, and then the solution was heated to 100 °C for drying. Subsequently, 6 mL oleic acid (OA) and 22.5 mL 1-octadecence (ODE) were added into the flask, then heated to 140 °C under vacuum until the solution get clear, after which it was cooled down to the room temperature. Then 15 mL methanol solution containing 6 mmol (0.15 g) NH_4F and 3.75mmol (0.233 g) NaOH were added dropwise, and it was vigorously stirred at the temperature of 50 °C overnight (12~15 h). Turn up the temperature to 70 °C~80 °C to evaporate most of methanol and then remove the methanol completely under vacuum. The next step is to heat the solution rapidly to 250 °C (~10 °C/min). After being stirred at 250 °C for 10 minutes under a gentle argon flow, the temperature was turned down to room temperature. Then the $NaGdF_4$ ND@OAs were precipitated using ethanol, centrifuged (10000 rpm, 10 min), and purified 3 times with ethanol, finally dispersed in 5 mL cyclohexane.

2 Additional Figures

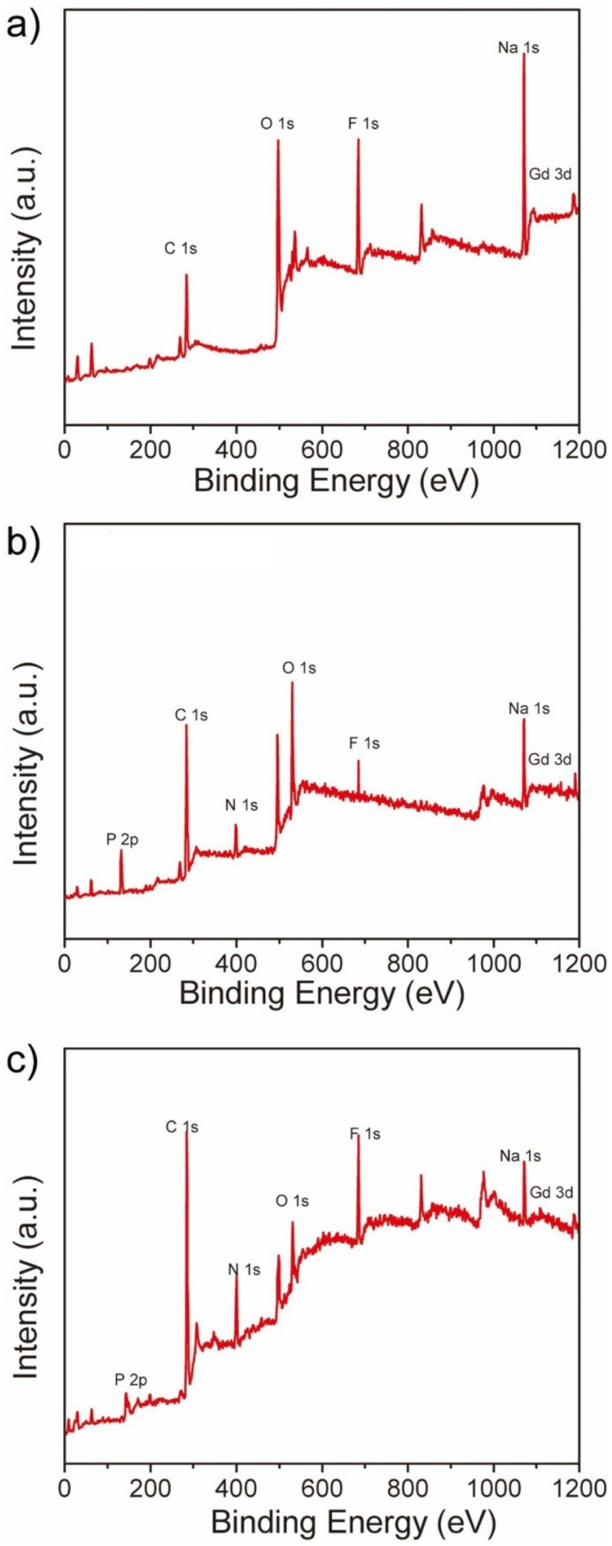

Figure S1 The XPS spectra of (a) $NaGdF_4$ ND@OAs, (b) $NaGdF_4$ ND@tryptone and (c) $NaGdF_4$ ND@HAs, respectively.

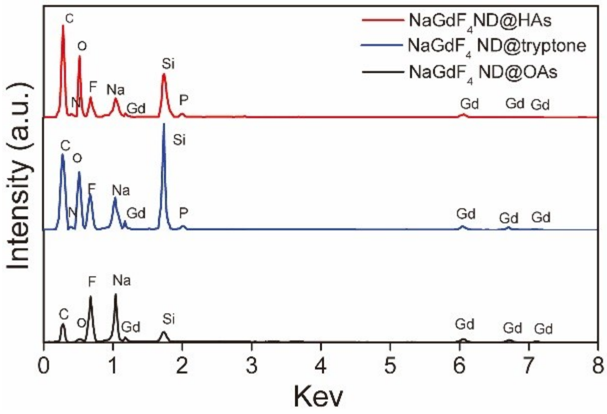

Figure S2 The EDS analysis of $NaGdF_4$ ND@OAs, $NaGdF_4$ ND@tryptone and $NaGdF_4$ ND@HAs, respectively.

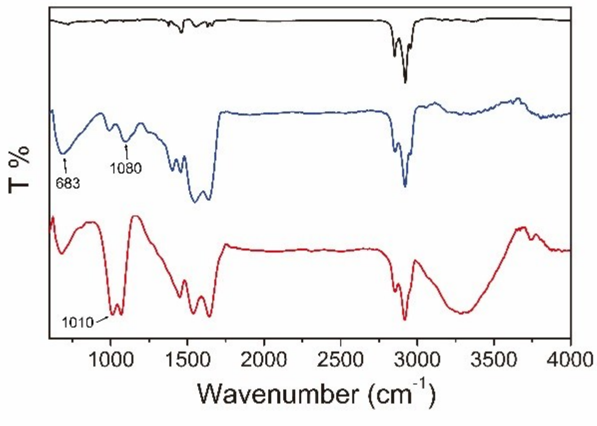

Figure S3 The FTIR of $NaGdF_4$ ND@OAs (black line), $NaGdF_4$ ND@tryptone (blue line) and $NaGdF_4$ ND@HAs (red line), respectively.

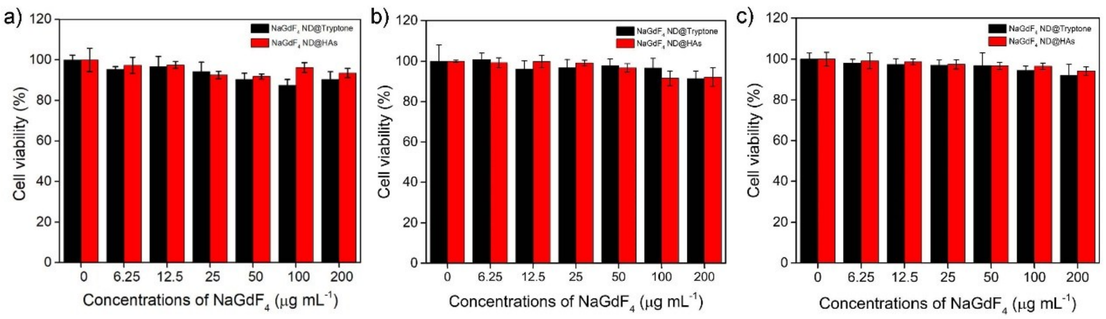

Figure S4 (a) MDA- MB-231, (b) MCF-7 and (c) 293 cell viabilities as a function of concentrations of $NaGdF_4$ ND@tryptone or $NaGdF_4$ ND@HAs. Error bars mean standbard deviations (n=5).

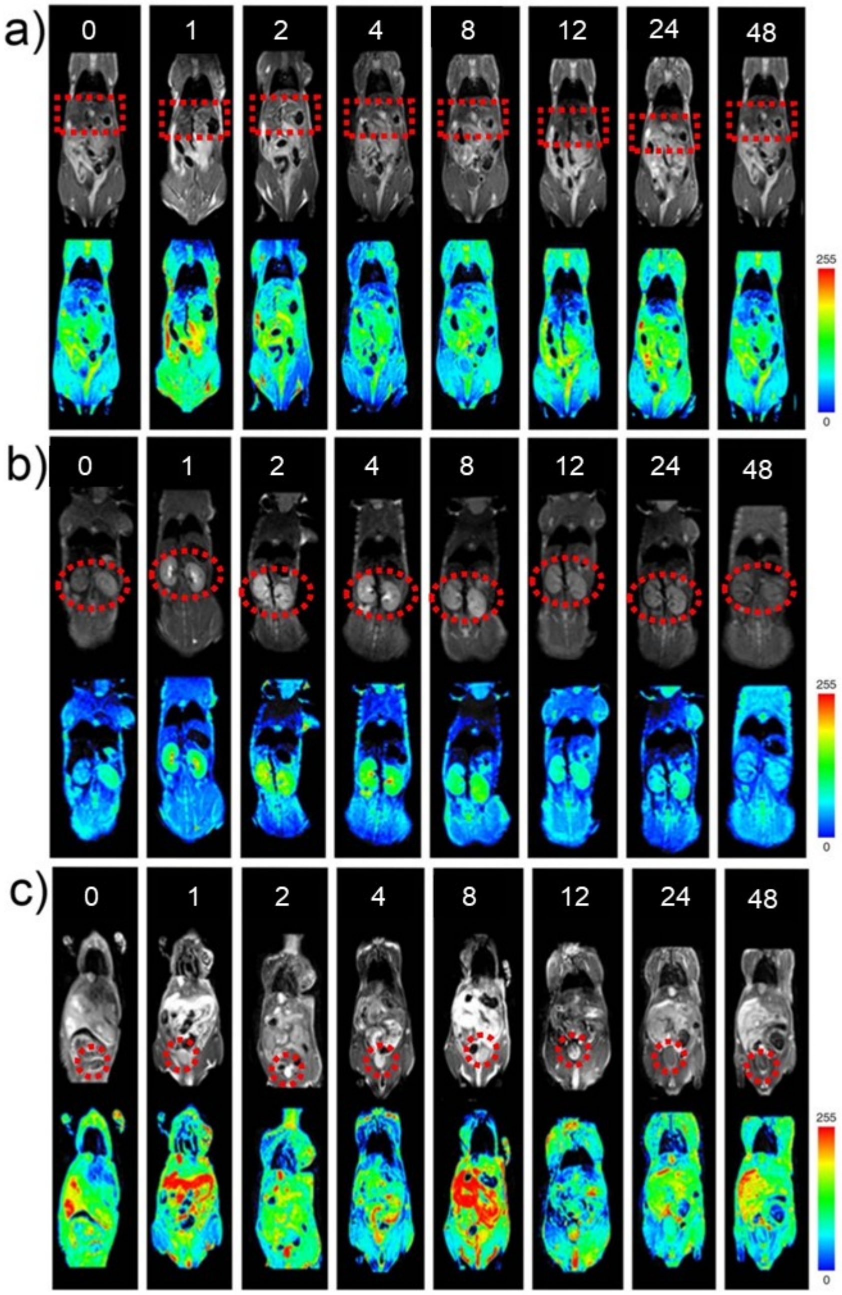

Figure S5 In vivo MR images of (a) liver, (b) kidney and (c) bladder of healthy Balb/c mice after intravenous injection of $NaGdF_4$ ND@HAs at different timed intervals (0 (pre-injection),1, 2, 4, 8, 12, 24 and 48 h) of post-injection, respectively.

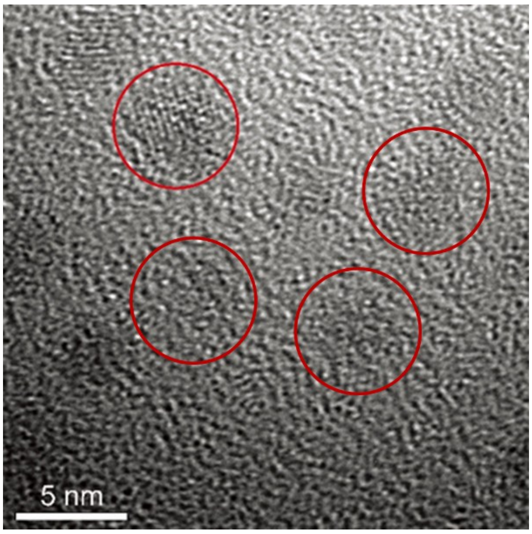

Figure S6 TEM micrograph of $NaGdF_4$ ND@HAs which were found in urine of the mouse.

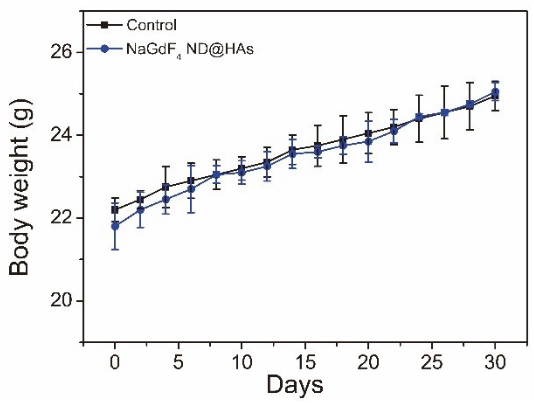

Figure S7 The body weights of healthy Balb/c mice (n = 3) after treated with 0.9 wt% NaCl solution (control) and 10 mg Gd kg^{-1} body $NaGdF_4$ ND@HAs, respectively.

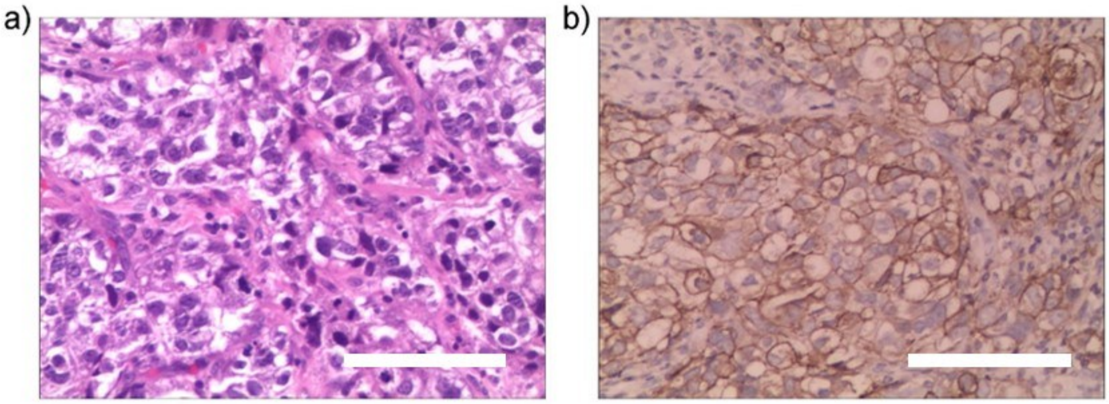

Figure S8 (a) H&E staining and (b) anti-CD44v6 staining section of tumor tissue from the mice. The tumor cells were fully stained by the anti-CD44v6 antibody. The scale bars are 200 μm.

3 Additional Tables

Table S1. The blood hematology analysis of healthy mouse at 1-day and 30-day post-injection of $NaGdF_4$ ND@HAs (10 mg Gd kg^{-1} body).

Hematological	Units	Control	1 d	30 d
WBC	$\times 10^9$/L	3.87 ±0.24	3.70 ±0.28	3.56 ±0.30
Neu	%	25.00 ±2.03	26.00 ±2.83	24.00 ±2.36
LY	%	68.21 ±3.48	68.35 ±5.25	69.22 ±4.83
RBC	$\times 10^{12}$/L	9.40 ±0.47	10.03 ±0.50	9.55 ±0.44
HGB	g/L	155.00 ±10.50	151.00 ±8.79	160.00 ±7.58
HCT	L/L	0.49 ±0.03	0.52 ±0.03	0.49 ±0.04
MCV	fL	51.70 ±5.82	52.80 ±4.62	50.90 ±4.79
MCH	pg	16.70 ±1.44	13.90 ±1.95	16.80 ±0.83
MCHC	g/L	351.00 ±8.55	329.80 ±11.56	332.90 ±15.0
RDW-CV	%	15.70 ±0.77	15.80 ±0.83	16.20 ±0.81
RDW-SD	fL	27.50 ±1.23	27.10 ±1.35	28.00 ±1.40
PLT	$\times 10^9$/L	430.00 ±50.51	457.00 ±44.99	439.00 ±45.95
MPV	fL	6.90 ±0.53	5.90 ±0.69	6.70 ±0.68
PDW	fL	15.20 ±1.27	15.70 ±0.98	15.40 ±1.12
PCT	%	0.29 ±0.01	0.29 ±0.01	0.30 ±0.01

4 Additional References

[S1] N. J. J. Johnson, W. Oakden, G. J. Stanisz, R. S. Prosser and F. C. J. M. van Veggel, *Chem. Mater.*, 2011, **23**, 3714-3722.

[S2] F. Liu, X. He, J. Zhang, H. Zhang and Z. Wang, *Small*, 2015, **11**, 3676-3685.

[S3] H. Chen, X. Li, F. Liu, H. Zhang, Z. Wang., *Mol. Pharmaceutics*, 2017, **14**, 3134−3141.
